# Identification and Biological Control of *Pestalotiopsis microspora* Causing Leaf Spot Disease on *Amomum villosum*

**DOI:** 10.3390/jof12080570

**Published:** 2026-08-01

**Authors:** Na Pu, Rui Wang, Wanshan Shao, Tangjie Zhao, Xiaoxuan He, Dan Li, Xiahong He, Xin Hao, Jie Chen

**Affiliations:** 1 Yunnan Provincial Key Laboratory for Conservation and Utilization of In-Forest Resource, Southwest Forestry University, Kunming 650224, China; pn18288426219@swfu.edu.cn (N.P.); 15721824391@163.com (R.W.); 13508825224@163.com (W.S.); hexiaoxuan2026@163.com (X.H.); lidan_163@163.com (D.L.); hxh@swfu.edu.cn (X.H.); 2 Wenshan State Research Institute of Forestry and Grassland Science, Wenshan 663099, China; zhaotangjie042@126.com

**Keywords:** leaf spot disease, *Amomum villosum*, *Pestalotiopsis microspora*, biological control

## Abstract

*Amomum villosum* is an evergreen perennial herb widely distributed in tropical and subtropical regions, with significant economic and medicinal importance. Throughout the cultivation process, it faces severe fungal disease problems that significantly impact its yield and quality. In July 2025, leaf spot disease with a 56% incidence was observed on *A. villosum* in Wenshan, Yunnan, China. Affected leaves initially developed irregular grayish-white lesions surrounded by brown margins. As symptom development progressed, semi-submerged black spots formed on the lesion surfaces. Severe infections resulted in premature leaf abscission and, ultimately, death of the entire plant. To identify the pathogen responsible, we conducted isolation and pathogenicity studies. Through morphological characterization, phylogenetic analysis (*ITS*, *LSU*, and *TUB*), and pathogenicity tests, *Pestalotiopsis microspora* was determined as a pathogen. Koch’s postulates were fulfilled on attached leaves. After 15 days, typical necrotic lesions appeared on inoculated leaves, while controls remained symptom-free. This is the first report of *A. villosum* leaf spot caused by *P. microspora* in China. Biocontrol assays revealed that *Trichoderma harzianum* T15 and *T. asperellum* T16 exhibited significant antagonistic activity against *P. microspora*, with inhibition rates of 44.77% and 50.70%, respectively. In addition, *Bacillus velezensis* SWFU41, isolated from healthy *A. villosum* leaves, showed superior disease suppression, achieving a control efficacy of 60.52%. Compared with the fungal biocontrol agents, *B. velezensis* demonstrated greater inhibitory activity against the pathogen, suggesting that bacterial antagonists may provide a more effective biological control strategy for managing *A. villosum* leaf spot disease. This is the first report of *P. microspora*-caused leaf spot disease on *A. villosum*. Furthermore, the comparative evaluation of fungal and bacterial antagonists demonstrated that *B. velezensis* SWFU41 outperformed *T. harzianum* and *T. asperellum* in disease suppression, highlighting its potential as a promising biocontrol agent for sustainable disease management. These findings provide a scientific basis for pathogen monitoring, epidemiological studies, and the development of integrated biological control strategies for *A. villosum* cultivation.

## 1. Introduction

*Amomum villosum* Lour. (Zingiberaceae) is an evergreen herbaceous plant [1]. Native to tropical and subtropical regions, it is primarily distributed in southern China, including Guangdong, Guangxi, Fujian, and Yunnan. As one of the traditional four major southern medicinal herbs, *A. villosum* has been used for over 1300 years in both medicine and food [2,3]. Modern medical research indicates that it has significant medicinal value in treating digestive and metabolic disorders. In particular, water extracts of *A. villosum* lower postprandial blood glucose and insulin levels, rendering them effective for the treatment of diabetes [4]. Its leaves and stems also hold potential as medicinal agents and dietary supplements for the prevention and treatment of diseases associated with inflammation and oxidative stress [5]. With increasing recognition of its medicinal and economic value, market demand for *A. villosum* has risen substantially. However, the increasing incidence of diseases threatens germplasm conservation, standardized cultivation, and the long-term viability of the industry. Among the diseases affecting *A. villosum*, leaf spot disease and anthracnose are the most destructive. Leaf spot disease is primarily associated with *Epicoccum thailandicum* [6] and *Pyricularia costina* [7], whereas anthracnose is caused by *Colletotrichum fructicola* [8], all of which result in significant economic losses. Therefore, timely disease detection, accurate pathogen identification, and effective management strategies are essential for improving crop yield and quality and for promoting the sustainable development of the *A. villosum* industry.

*Pestalotiopsis* belongs to the phylum Ascomycota, class Sordariomycetes, order Amphisphaeriales, and family Sporocadaceae [9]. This genus comprises a diverse group of fungi, many of which are known only from their asexual morphs, and it is widely distributed in tropical and temperate ecosystems [10,11]. Species of *Pestalotiopsis* were originally classified within the genus *Pestalotia* until Steyaert redefined the taxonomy in 1949 based on the number of median conidial cells, establishing *Pestalotiopsis* (five-celled conidia, type species *P. guepinii*), *Pestalotia* (six-celled conidia), and *Truncatella* (four-celled conidia) [12]. Species of *Pestalotiopsis* are recognized as important plant pathogens responsible for a wide range of diseases in numerous host species [13]. They primarily infect leaves, fruits, and stems, causing substantial economic losses in agricultural and horticultural crops [14,15], including *Castanea mollissima* leaf spot caused by *P. kenyana* [16], stem spot caused by *P. uvicola* [17], *Psidium guajava* (scab) [18], and canker and stem blight of various fruits [19]. In addition to their pathogenic roles, *Pestalotiopsis* species are important endophytic symbionts, with *P. funerea* representing the first species reported as an endophyte, isolated from *Sequoia sempervirens* [20]. The morphological classification of *Pestalotiopsis* spp. primarily relies on several key characteristics: conidial septation (number of cells and septa), the pigmentation of median cells, and the number and length of apical and basal appendages [11]. However, relying solely on traditional morphological studies is no longer sufficiently reliable. Consequently, the field has widely adopted molecular methods integrated with multigene phylogenetic analysis, thereby ensuring greater accuracy in species identification [21,22].

To control diseases caused by *Pestalotiopsis* species, various biological control agents have been investigated. Previous studies have demonstrated that *B. velezensis* CE100 exhibits strong antagonistic activity against leaf blight caused by *Pestalotiopsis maculans*. This strain produces cell wall-degrading enzymes, including chitinase, *β*-1,3-glucanase, and protease, which induce cell wall degradation and hyphal deformation, resulting in a 54.94% reduction in mycelial growth [23]. Similarly, *Paecilomyces lilacinus* A65 has been reported to suppress *Pestalotiopsis theae*, achieving an inhibition rate of 75.46% [24]. *Pestalotiopsis* species infect a broad range of fruits, cash crops, medicinal plants, and ornamental species, causing leaf spot and other destructive diseases. Their host range and associated economic impacts continue to expand, posing increasing threats to agricultural production and ecosystem stability. Leaf spot disease primarily impairs plant physiological functions by damaging photosynthetically active tissues. Following infection, lesions develop and disrupt mesophyll tissue structure, leading to reduced photosynthetic efficiency and diminished assimilate production. Consequently, fewer nutrients are available for plant growth and fruit development, resulting in reduced biomass accumulation, smaller fruit size, and lower crop productivity [25]. Consequently, studies on the epidemiology and management of diseases caused by *Pestalotiopsis* species are essential for safeguarding agricultural production and maintaining ecological sustainability.

Yunnan Province is the largest production region of *A. villosum* in China, with major cultivation areas concentrated in Xishuangbanna, Wenshan, and Honghe. As cultivation of *A. villosum* has expanded rapidly across Yunnan Province, disease pressure has intensified markedly. Nevertheless, the causal agents of emerging leaf diseases remain unidentified, and the biocontrol potential of indigenous antagonists has yet to be evaluated. The objectives of this study were to identify the pathogen responsible for a newly emerging leaf spot disease of *A. villosum* and to evaluate the efficacy of selected fungal and bacterial antagonists against the pathogen. Pathogen identification was conducted using morphological characterization, multilocus phylogenetic analyses, and pathogenicity tests. As the genera *Trichoderma* and *Bacillus* possess a broad spectrum of control efficacy, the biological control potential of two *Trichoderma* (*T. harzianum* T15 and *T. asperellum* T16) strains previously isolated by our research group and of an endophytic bacterium (SWFU41) isolated from healthy *A. villosum* leaves was assessed against the pathogen. This study provides the first evidence that *P. microspora* is the causal agent of *A. villosum* leaf spot disease in China and demonstrates the potential of native beneficial microorganisms, particularly *B. velezensis* SWFU41, as promising biological control agents. These findings contribute to the development of sustainable disease management strategies and provide a scientific basis for improving the health and productivity of *A. villosum* cultivation systems.

## 2. Materials and Methods

### 2.1. Sample Collection and Separation

From March 2024 to July 2025, a field survey of *A. villosum* leaf spot disease was conducted in a 1.6-ha plot in the forest understory in Maguan County, Wenshan Prefecture, Yunnan Province, China (103°35′ E, 22°40′ N). Using a five-point sampling method, a total of 120 plants were assessed, and leaves from the north, east, south, and west sides of each plant were examined for disease symptoms to determine disease incidence. The disease incidence rate is calculated using Equation (1).

I (%) = (ni/N) × 100%(1)

(ni: number of diseased plants examined, N: total number of plants examined).

Subsequently, 30 symptomatic leaves were randomly selected for microscopic examination using an Olympus CX33 microscope (Olympus, Tokyo, Japan). To isolate the causal pathogen, leaf tissues from the margins of typical lesions were surface sterilized, and small sections at the interface between diseased and healthy tissues were excised using a sterile scalpel. The tissue sections were sequentially immersed in 75% ethanol for 30 s and 1% sodium hypochlorite (NaClO) for 40 s, rinsed three times with sterile distilled water, and dried on sterile filter paper. The pathogen was isolated using the single spore isolation method [26]. Individual conidia were observed under a microscope, picked up with an entomological needle, and transferred onto potato dextrose agar (PDA; 200 g potato infusion, 20 g glucose, and 20 g agar L^−1^). The plates were incubated at 25 °C until individual colonies developed. Under aseptic conditions, hyphal tips from newly emerging colonies were transferred onto fresh PDA plates and incubated for 7 days at 25 °C in the dark. Twenty isolates with similar morphological characteristics were obtained. Among these isolates, SWFU30 was selected as a representative isolate based on its stable colony morphology and typical characteristics observed during purification. The isolate was deposited and maintained in the culture collection of Southwest Forestry University, Kunming, China.

To isolate endophytic bacteria, five healthy *A. villosum* leaves were collected from the same healthy three-year-old plant, rinsed thoroughly under running tap water, and surface-sterilized by sequential immersion in 75% ethanol for 1 min and 2.5% sodium hypochlorite for 3–5 min, followed by 3–5 rinses with sterile distilled water. The sterilized leaf tissues were homogenized in sterile saline, serially diluted (10^−1^–10^−5^), and 100 μL aliquots of dilution (10^−4^, 10^−5^) were spread onto Luria–Bertani (LB) agar plates (10 g/L tryptone, 5 g/L yeast extract, 10 g/L NaCl, and 15 g/L agar), five replicates for each concentration. The plates were incubated at 30 °C for 1–3 d. Colonies displaying distinct morphological characteristics were selected and purified by three successive streaking on fresh LB agar plates. After removing duplicate morphotypes, a total of 24 representative endophytic bacterial isolates were obtained. Based on preliminary antagonistic screening, isolate SWFU41 was selected for further experiments and preserved in 30% (*v*/*v*) glycerol at −80 °C. The remaining isolates were not retained [27]. SWFU41 was deposited and maintained in the culture collection of Southwest Forestry University, Kunming, China, under the accession number SWFU41.

### 2.2. Morphological Identification

Purified fungal isolates (SWFU30, SWFU31) were cultured on PDA plates at 25 °C for 7 days. Colony characteristics were recorded, and fungal structures were examined under an optical microscope (Olympus CX33, Tokyo, Japan). Conidia produced by the purified cultures were collected and observed microscopically. The length, width, and morphological characteristics of conidia were measured based on 50 randomly selected conidia (*n* = 50).

The biocontrol bacterium isolate SWFU41 was streaked onto LB agar plates and incubated at 30 °C for 24 h. Colony morphology was recorded, and gram staining was performed according to the manufacturer’s instructions (Hunan BKMMAM Holding Co., Ltd., Changde, Hunan, China).

### 2.3. Pathogenicity Testing

To confirm its pathogenicity, a representative preserved strain SWFU30, was selected and tested in accordance with Koch’s postulates [28]. Healthy three-year-old *A. villosum* plants without visible disease symptoms were used. A conidial suspension of each isolate (1 × 10^6^ conidia mL^−1^), with the concentration determined using a hemocytometer, was prepared for inoculation. Five potted plants were used for each treatment, and five fully expanded leaves per plant were selected for inoculation. A 10 μL aliquot of the conidial suspension was injected into the leaf tissue using a sterile microsyringe, whereas control plants were inoculated with an equal volume of sterile distilled water [29]. Twenty-five leaves were used for each treatment. Following inoculation, plants were maintained under high humidity conditions using polyethylene bags for 72 h and subsequently transferred to a growth chamber at 25 °C under a 12 h light/12 h dark photoperiod. Symptom development was assessed 15 d after inoculation. To complete Koch’s postulates, the pathogen was re-isolated from newly developed lesions using the single-spore isolation method. The recovered isolate (SWFU31) was compared with the original isolate (SWFU30) based on colony morphology, conidial characteristics, and sequence analyses of the *ITS*, *LSU*, and *TUB* gene regions.

### 2.4. DNA Extraction and Polymerase Chain Reaction Sequencing

Mycelial plugs (5 mm in diameter) were cultured on potato dextrose agar (PDA) plates at 25 °C for 5 days. Three actively growing plugs were transferred to 100 mL of potato dextrose broth (PDB; 200 g potato extract and 20 g dextrose L^−1^) and incubated at 25 °C in the dark on a rotary shaker at 120 rpm for 8 days. Approximately 100 mg of fresh mycelial biomass was harvested, immediately frozen in liquid nitrogen, and ground into a fine powder. Genomic DNA was extracted using a Genomic DNA Kit according to the manufacturer’s instructions (Tiangen, DP305, Beijing, China).

The bacterial isolates were inoculated onto Luria–Bertani (LB) agar plates and incubate at 28 °C for 24–48 h. Pick individual colonies and transfer them to 10 mL of LB liquid medium. Incubate at 28 °C with shaking at 180 rpm for 12–16 h. Genomic DNA from bacterial isolates was extracted using the cetyltrimethylammonium bromide (CTAB) method [30]. DNA quality and integrity were assessed by electrophoresis on 0.8% agarose gels. The internal transcribed spacer (*ITS*), large subunit ribosomal RNA gene (*LSU*), *β*-tubulin gene (*TUB*) for fungi, 16S rRNA gene, and *gyrB* gene for bacteria were amplified using the corresponding primer pairs listed (Table 1). PCR amplifications were performed in a 50 μL reaction mixture containing 25 μL Premix Taq™ (TaKaRa Taq™ Version 2.0 Plus Dye; RR901A, Takara, Dalian, China), 1 μL DNA template, 2 μL each of the forward and reverse primers, and sterile double-distilled water to a final volume of 50 μL. The amplification program consisted of an initial denaturation at 94 °C for 2 min, followed by 35 cycles of denaturation at 94 °C for 30 s, annealing at the temperatures specified for each primer pair in Table 1, and extension at 72 °C for 2 min, with a final extension at 72 °C for 10 min. All primers were synthesized by Sangon Biotech Co., Ltd. (Shanghai, China). PCR products were verified by agarose gel electrophoresis and subsequently sequenced using the Sanger sequencing method by Sangon Biotech Co., Ltd. (Shanghai, China).

### 2.5. DNA Sequence Analysis

The obtained sequences were compared with reference sequences available in the NCBI GenBank database using BLASTN 2.17.0 (https://blast.ncbi.nlm.nih.gov/). The sequences were aligned using ClustalW implemented in MEGA 12 [37]. Phylogenetic relationships were inferred through the Maximum Likelihood method with tamura 3-parameter model and gamma distributed with invariant sites (G + I). Bootstrap support values were estimated with 1000 replicates to assess the reliability of tree branches [38]. All sequence data from this study have been submitted to the GenBank database.

### 2.6. Antifungal Activity Tests

Two *Trichoderma* strains, *T. harzianum* T15 and *T. asperellum* T16, were obtained from the strain collection of the Forest Disaster Warning and Control Laboratory, Southwest Forestry University, Kunming, China. The antagonistic activity of *Trichoderma* strains against *P. microspora* was evaluated using a dual-culture assay [39]. A 5 mm-diameter mycelial plug of *P. microspora* and a 5 mm-diameter plug of each *Trichoderma* strain were placed on opposite sides of PDA plates, approximately 3.5 cm from the center. Plates inoculated only with the pathogen served as controls. Each treatment included five replicates and was incubated at 28 °C. Colony diameters were measured when the colonies of the pathogen and antagonist contacted each other. The inhibition rate was calculated using Equation (2).

Inhibition rate (%) = [(D − d)/D] × 100%(2)

D: Fungal colony diameter in the control group (cm); d: Fungal colony diameter in the experimental group (cm).

The antagonistic activity of *B. velezensis* SWFU41 against *P. microspora* was evaluated using a four-point confrontation assay [40]. A 5 mm-diameter mycelial plug of the pathogen was placed at the center of a PDA plate, and four sterile filter paper discs (6 mm diameter) were positioned 3 cm from the center. Each disc was inoculated with 5 μL of a 12 h-old bacterial culture grown in LB broth. Sterile LB broth was used as the negative control. Plates were incubated at 28 °C, and colony diameters were recorded every 24 h. Five replicates were performed for each treatment, and the inhibition rate was calculated using the equation described above.

### 2.7. Statistical Analysis

Data are presented as the mean ± standard deviation (SD). Statistical analyses were performed using GraphPad Prism 9.0 (GraphPad Software, San Diego, CA, USA). Prior to ANOVA, data normality and homogeneity of variances were assessed using the Shapiro–Wilk test and Levene’s test, respectively. Differences among treatments were analyzed using one-way analysis of variance, followed by Tukey’s multiple comparison test. Statistical significance was determined at *p* < 0.05.

## 3. Results

### 3.1. Sample Collection

**Figure 1:**
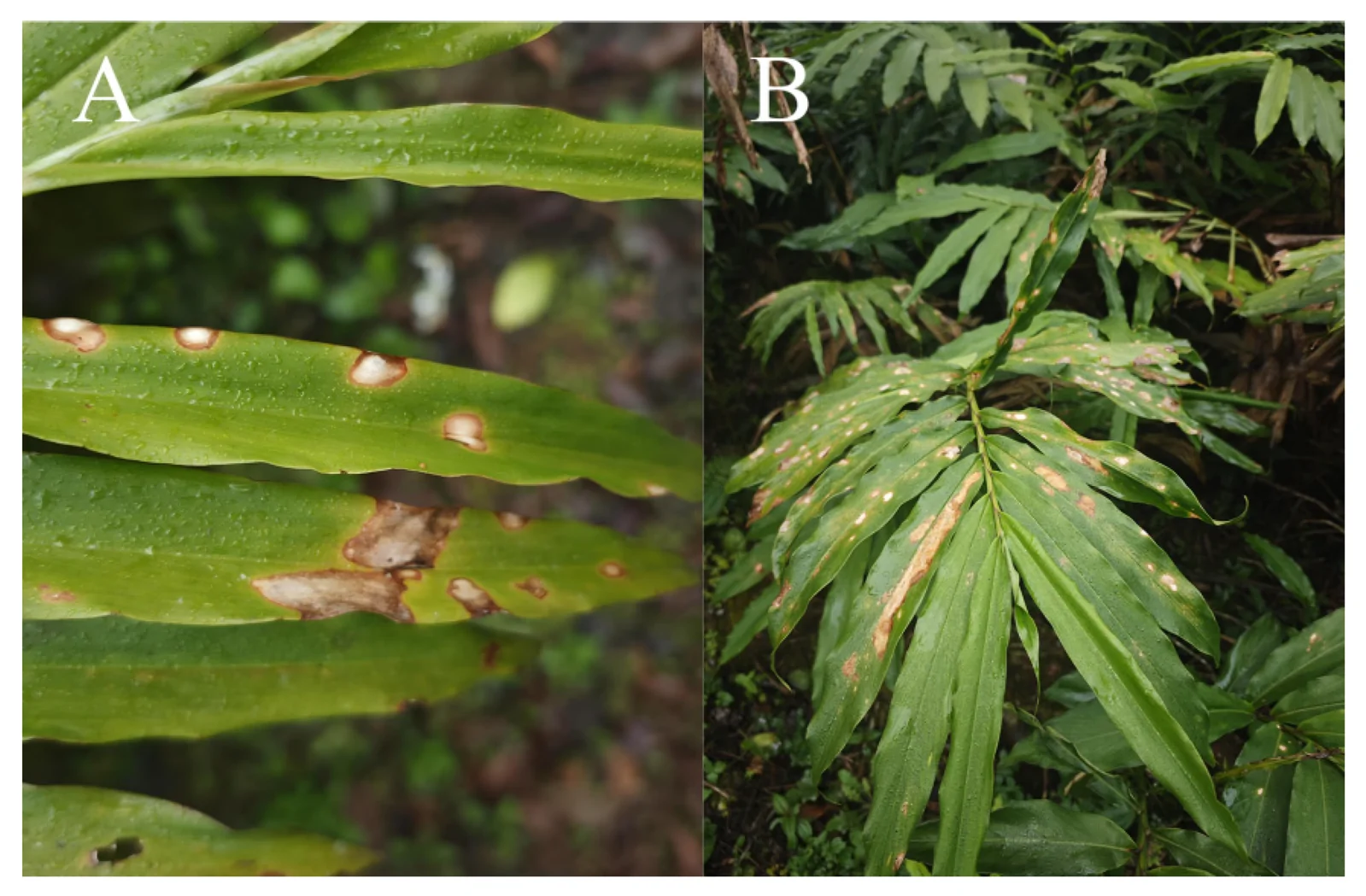
Symptoms of *A. villosum* leaf spot observed in the field. (**A**) Typical lesions on infected leaves; (**B**) Diseased plants in the field.

In July 2025, leaf samples exhibiting leaf spot symptoms were collected from *A. villosum* plantations in Maguan County, Wenshan Prefecture, Yunnan Province, China, and disease occurrence was investigated. Disease incidence reached 56% in the surveyed plantations. Affected leaves initially developed irregular grayish-white lesions with brown margins (Figure 1A,B). As disease progression continued, semi-submerged black acervuli formed on the lesion surfaces. Severe infections resulted in premature leaf abscission and, in some cases, death of the entire plant.

### 3.2. Morphological Characteristics

**Figure 2:**
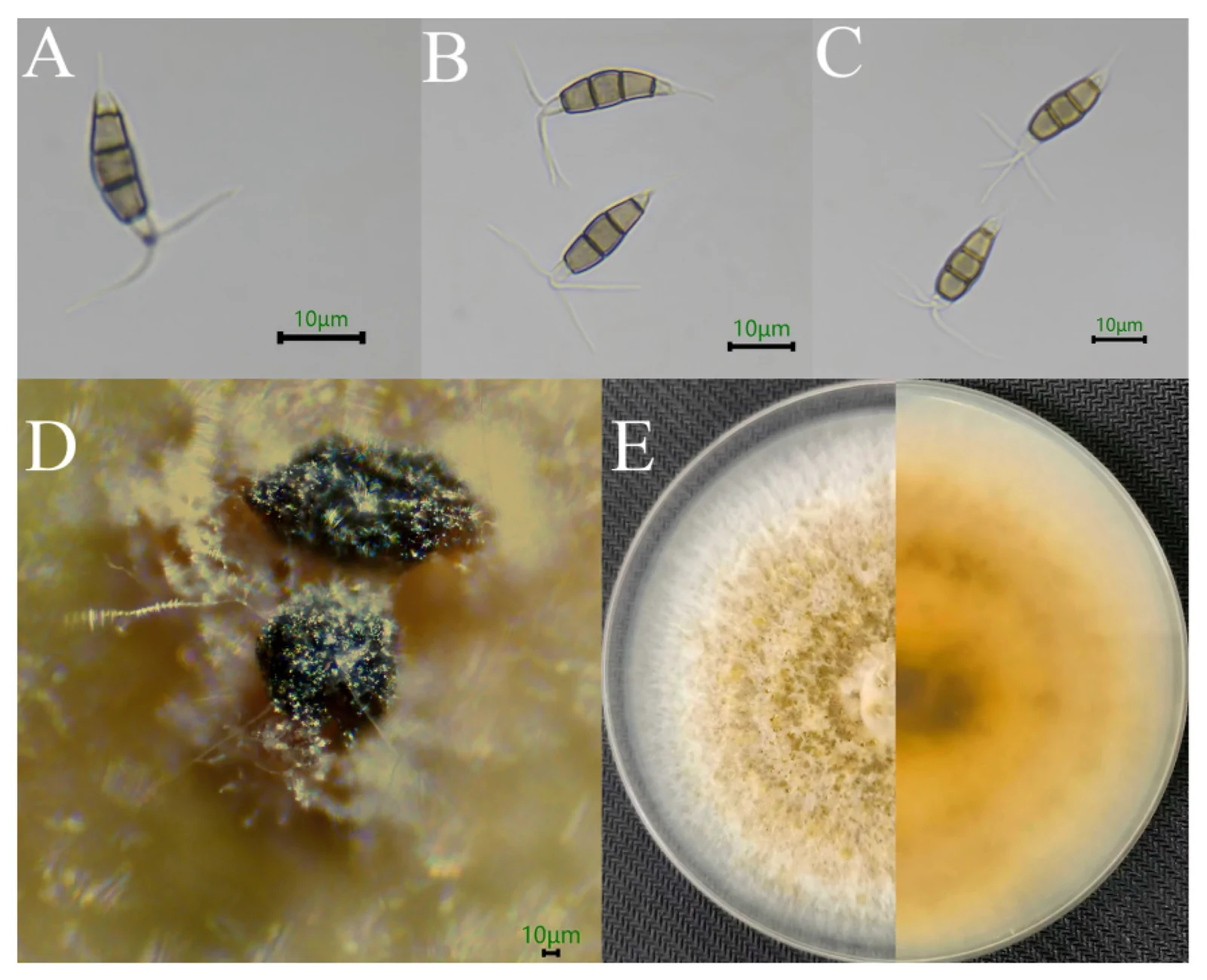
Morphological characteristics of *P. microspora*. (**A**–**C**) Conidia; (**D**) Acervuli; (**E**) Colony on PDA after 15 d, scale bar = 10 μm.

A total of 20 fungal isolates were obtained from diseased *A. villosum*, and all exhibited similar morphological characteristics on PDA. As a representative isolate, the morphological description of the deposited strain SWFU30 is presented below. Conidia were fusiform, slightly curved, 5-celled, and measured 21.0–27.3 × 5.3–7.1 μm. The apical cell was hyaline and bore 2–4 apical appendages measuring 9.1–15.7 μm in length. The three median cells were pale brown to light brown, slightly constricted at the septa, and measured 14.2–18.2 μm in total length. The basal cell was hyaline, conical, and possessed a single basal appendage measuring 3.0–7.0 μm in length (Figure 2A–C). Acervuli were black, subepidermal to semi-immersed, and scattered on the lesion surface. Mature acervuli ruptured through the epidermis and released masses of conidia under humid conditions. Colonies on PDA were initially white and became yellowish with age, exhibiting dense aerial mycelia (Figure 2E). After 15 d of incubation at 25 °C, colonies covered the entire plate and produced abundant black acervuli (Figure 2D). Based on these morphological characteristics, the isolate was preliminarily identified as *P. microspora*.

### 3.3. Pathogenicity Test

**Figure 3:**
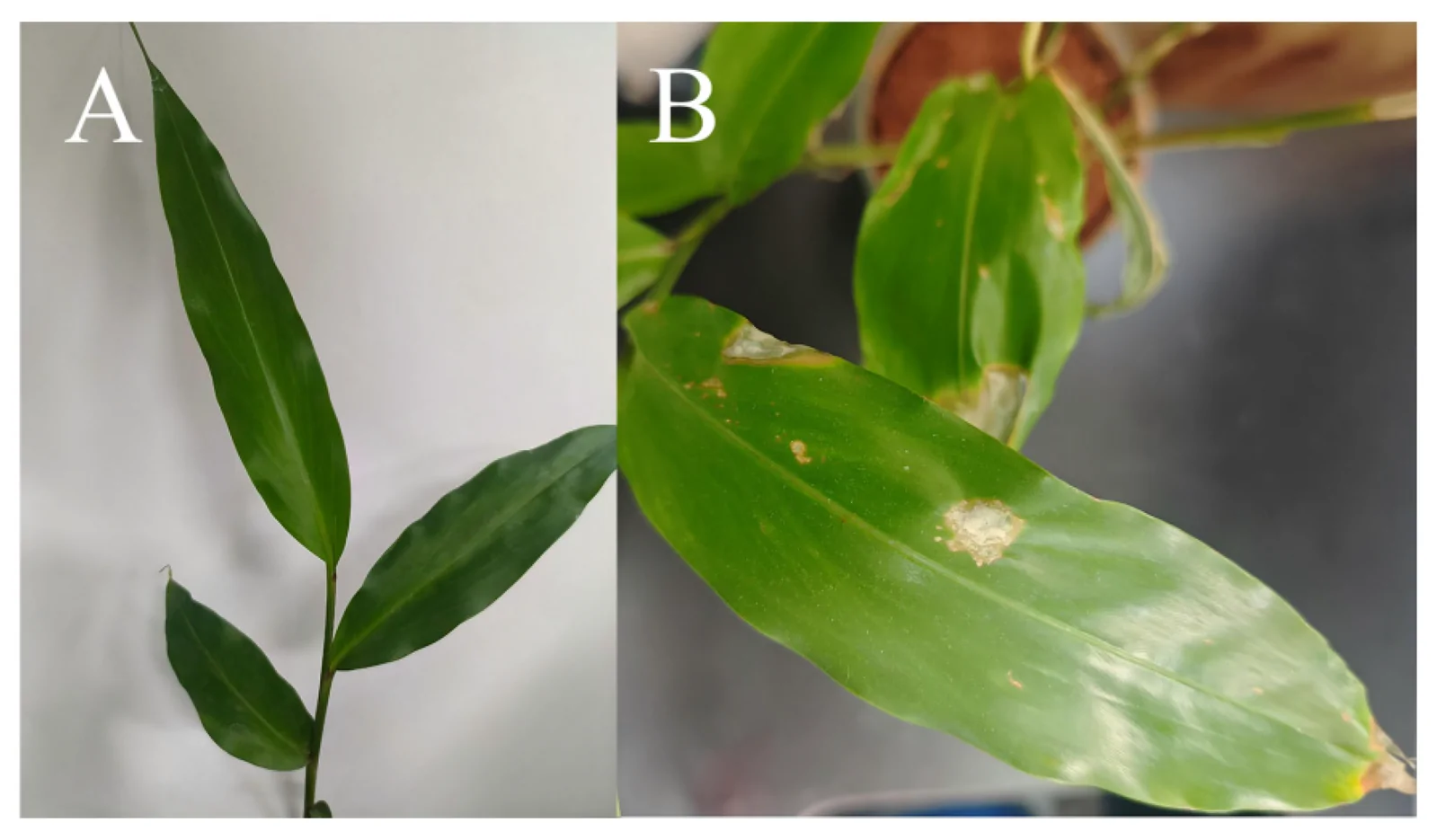
Symptoms of *P. microspora* and symptoms of re-inoculated leaves after 15 d and sterile water processing. (**A**) Control; (**B**) Re-inoculated.

Fifteen days after inoculation, typical leaf spot symptoms developed on the inoculated leaves and were consistent with those observed under natural field conditions. Symptoms initially appeared as irregular grayish-white lesions with brown margins and gradually expanded over time (Figure 3B). In contrast, no symptoms developed on the control leaves treated with sterile water (Figure 3A). Isolate SWFU30 was selected as the representative isolate for pathogenicity testing. The strain SWFU31 was re-isolated from the symptomatic leaves. Based on morphological characteristics and subsequent molecular identification, the re-isolated strain SWFU31 was confirmed as the same pathogen as the original isolate SWFU30. The recovered isolate SWFU31 exhibited colony morphology, conidial characteristics, and sequence data of *ITS*, *LSU*, and *TUB* identical to those of the original isolate SWFU30.

### 3.4. Phylogenetic Analysis

Partial sequences of the internal transcribed spacer (*ITS*), *β*-tubulin (*TUB*), and large subunit ribosomal RNA gene (*LSU*) were amplified. The obtained sequences were subjected to BLAST 2.17.0 (https://blast.ncbi.nlm.nih.gov/) searches against the NCBI GenBank database. The *ITS*, *TUB*, and *LSU* sequences showed 99.84%, 99.79%, and 100% identity, respectively, to corresponding reference sequences of *P. microspora* (accession numbers KY366172, JN314419, and KY366173), indicating that the isolate belonged to the genus *Pestalotiopsis* and was closely related to *P. microspora*.

**Figure 4:**
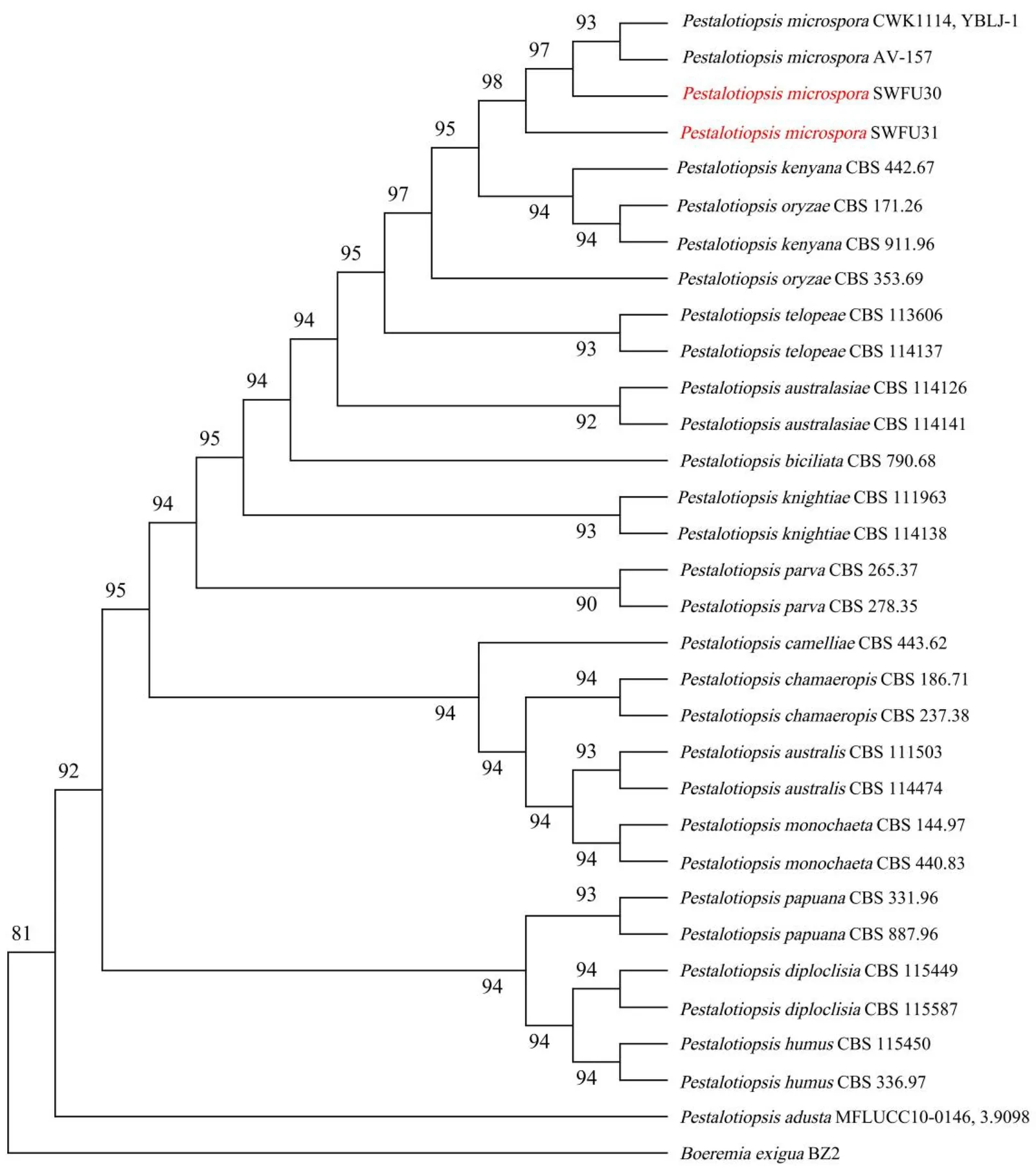
Maximum Likelihood phylogenetic tree inferred from combined *ITS*, *LSU*, and *TUB* sequence data. Bootstrap support values (%) based on 1000 replicates are shown above the branches. Strains obtained in this study are highlighted in red.

The generated sequences from (SWFU30 and SWFU31) were deposited in GenBank under accession numbers PX806344 and PX810979 (*ITS*), PZ224677 and PZ224678 (*TUB*), and PX806352 and PX810981 (*LSU*). To clarify the phylogenetic position of the isolates, a multilocus phylogenetic analysis was conducted using concatenated *ITS*, *TUB*, and *LSU* sequences. The dataset comprised 96 reference sequences representing 17 *Pestalotiopsis* taxa retrieved from GenBank (Table 2). Phylogenetic tree revealed that isolates SWFU30 and SWFU31 clustered together with authenticated reference strains of *P. microspora*, forming a distinct clade with high bootstrap support (Figure 4). No obvious genetic divergence was observed between the two isolates and other representative strains of *P. microspora*. The phylogenetic placement of the isolates was consistent with their morphological characteristics, including colony morphology, conidial dimensions, and appendage structure. Therefore, based on both morphological observations and multilocus phylogenetic evidence, they were identified as *P. microspora*.

### 3.5. Antifungal Activity of Trichoderma *spp.*

The antagonistic activity of *T. asperellum* and *T. harzianum* against *P. microspora* was evaluated using a dual-culture confrontation assay. Both antagonists significantly suppressed the mycelial growth of *P. microspora* compared with the control throughout the incubation period.

**Figure 5:**
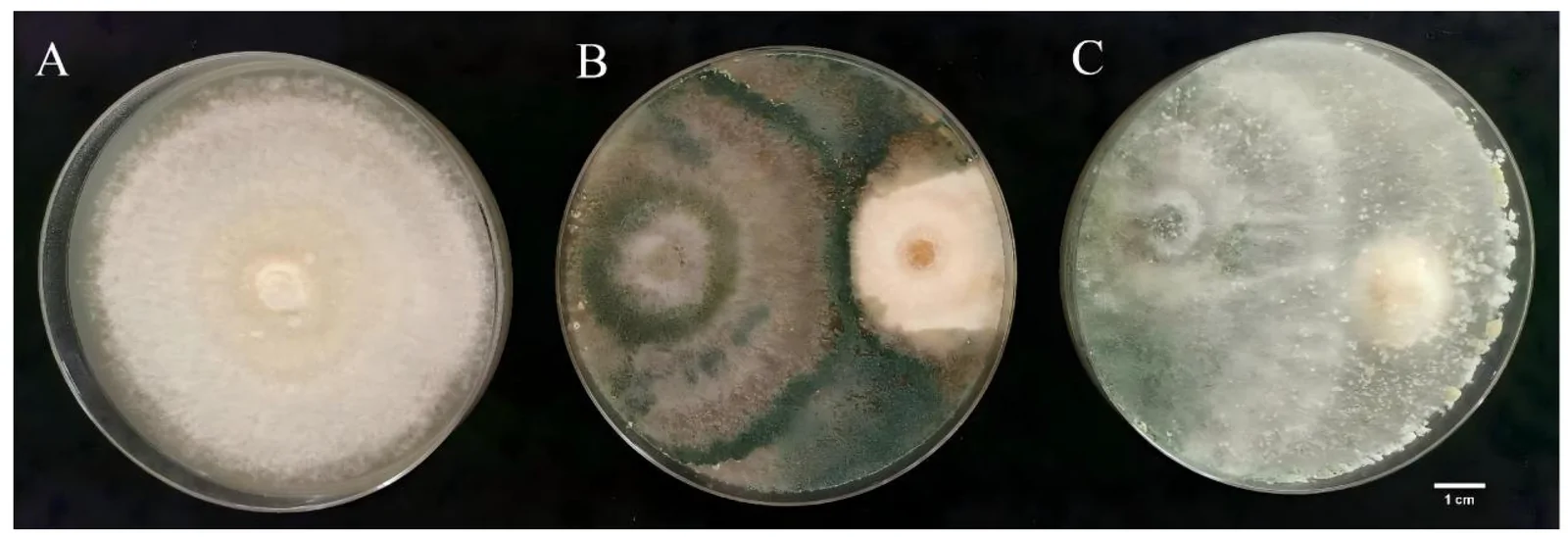
Inhibition of *Trichoderma* spp. against *P. microspora*. (**A**) Control; (**B**) *T. harzianum*; (**C**) *T. asperellum*.

The inhibitory effect increased progressively with culture time. For *T. asperellum*, the inhibition rate increased from 7.76 ± 2.10% on 3 d to 50.70 ± 2.41% on 7 d (Figure 5A,C), whereas *T. harzianum* exhibited inhibition rates ranging from 7.02 ± 3.04% to 44.77 ± 3.58% over the same period (Table 3) (Figure 5A,B). Throughout the experiment, *T. asperellum* consistently exhibited numerically higher inhibition rates than *T. harzianum* at all measured time points (Table 3). After 7 d of incubation, both *Trichoderma* species had colonized the entire PDA plate. However, *T. asperellum* showed a higher numerical inhibition level against *P. microspora* than *T. harzianum* under the same conditions.

### 3.6. Identification and Antagonistic Activity of Strain SWFU41

**Figure 6:**
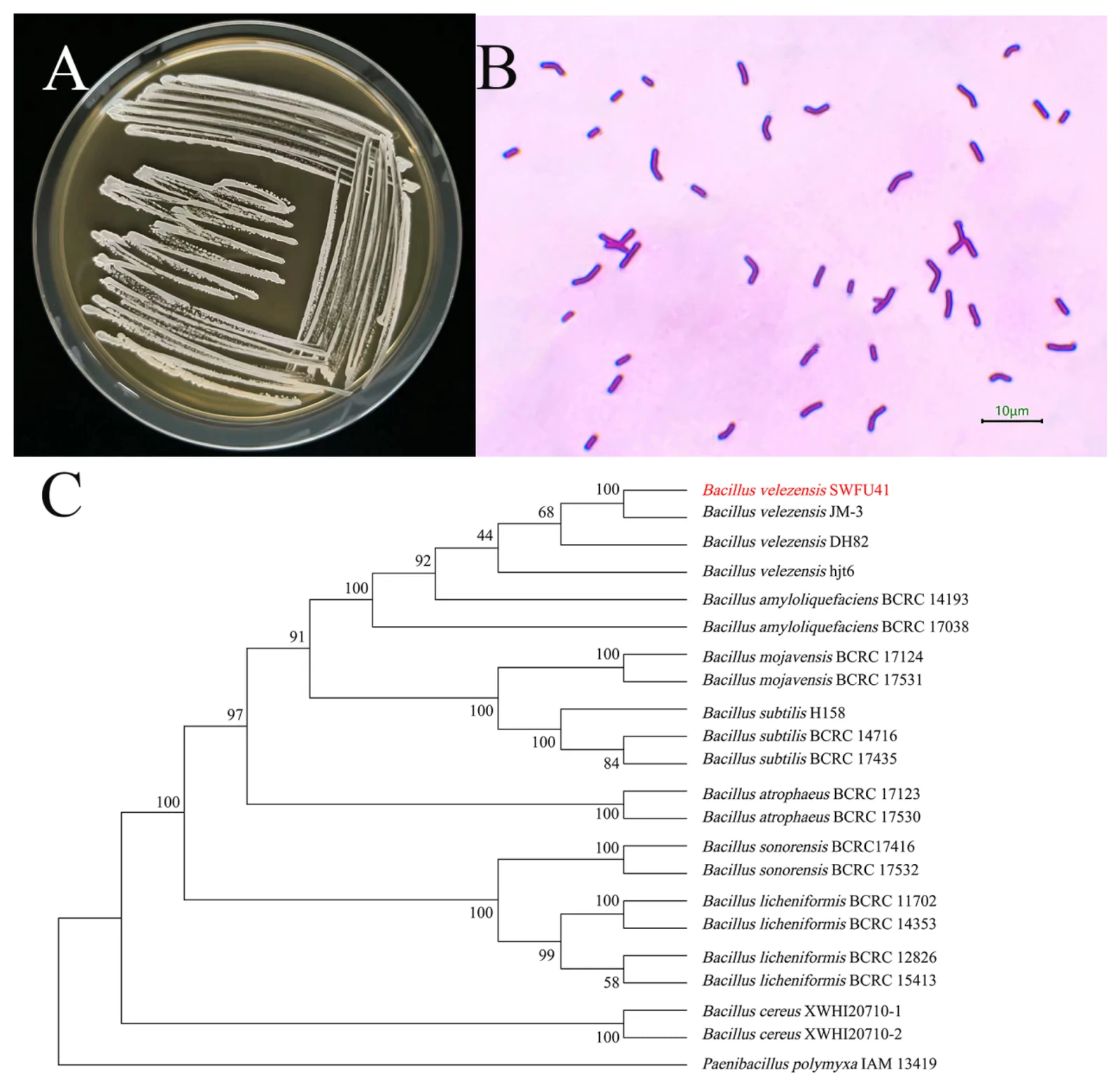
Morphological and molecular identification of strain SWFU41. (**A**) Colony morphology on LB agar; (**B**) Gram-stained spores, scale bar = 10 μm; (**C**) Maximum Likelihood phylogenetic tree based on concatenated 16S rRNA and *gyrB* sequences. Bootstrap values (%) from 1000 replicates are shown at the nodes. The isolate obtained in this study is indicated in red.

Among the bacterial isolates obtained from healthy *A. villosum* leaves, SWFU41 showed strong antagonistic activity against *P. microspora* and was selected for identification and biocontrol evaluation. Strain SWFU41 was cultured on LB agar at 30 °C for 2 d. Colonies were circular, smooth, moist, and convex, with entire to slightly irregular margins and white to pale-yellow pigmentation (Figure 6A). Gram staining revealed that the cells were Gram-positive rods (Figure 6B). These morphological characteristics were consistent with those commonly reported for *Bacillus* species. To further clarify its taxonomic identity, partial 16S rRNA and *gyrB* gene sequences were analyzed.

The obtained sequences were deposited in GenBank under accession numbers PZ498242 (16S rRNA) and PZ498543 (*gyrB*). BLAST 2.17.0 (https://blast.ncbi.nlm.nih.gov/) analysis showed that the 16S rRNA and *gyrB* sequences shared 100% identity with corresponding *B. velezensis* reference sequences (ON413870 and MT999486), indicating a close relationship between SWFU41 and *B. velezensis*. To confirm its phylogenetic placement, a multilocus phylogenetic analysis was performed using concatenated 16S rRNA and *gyrB* sequences. The dataset comprised 21 reference sequences representing 9 *Bacillus* taxa retrieved from GenBank (Table 4). The phylogenetic tree revealed that strain SWFU41 clustered with authenticated reference strains of *B. velezensis* and formed a well-supported clade with high bootstrap values (Figure 6C). Therefore, strain SWFU41 was identified as *B. velezensis* and was subsequently evaluated for its antagonistic activity against *P. microspora*.

**Figure 7:**
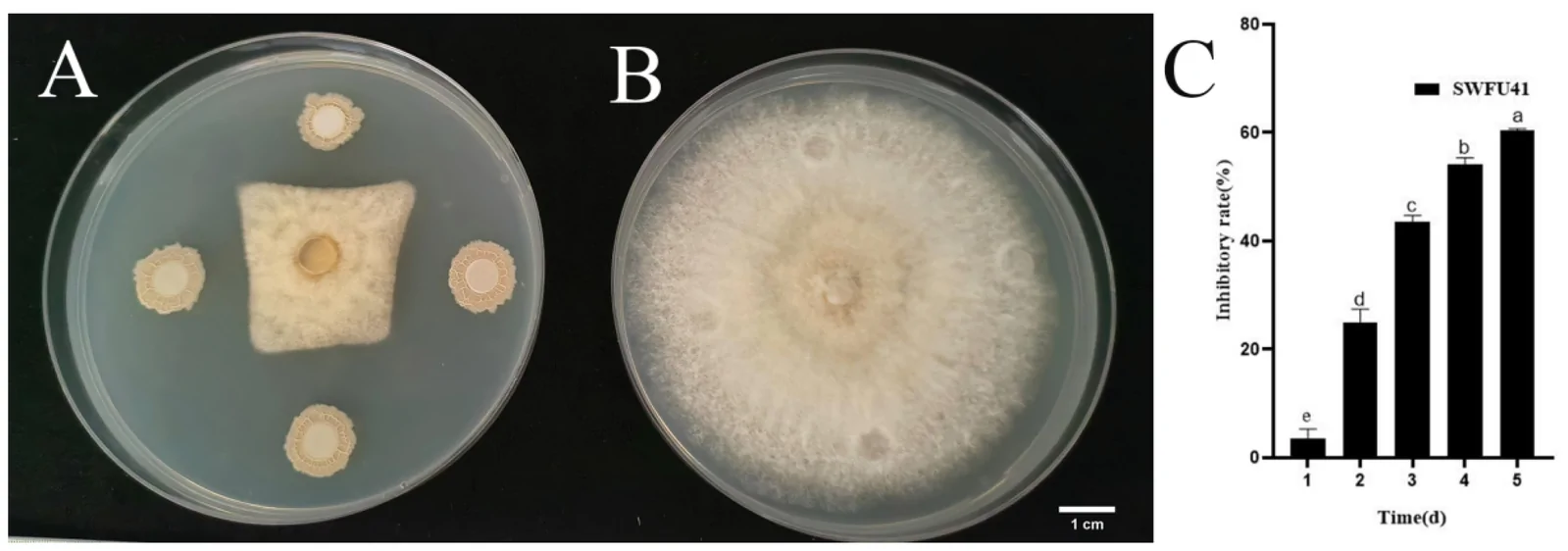
Antagonistic activity of strain SWFU41 against *P. microspora*. (**A**) Treatment at 5 d, (**B**) Control at 5 d, (**C**) The inhibitory rates of SWFU41 against *P. microspora*. Different letters above the bars indicate significant differences in inhibition rates among incubation times (*p* < 0.05).

The antagonistic activity of strain SWFU41 against *P. microspora* was evaluated using a four-point confrontation assay. Compared with the control, SWFU41 effectively suppressed the growth of *P. microspora*, and the inhibition rate increased progressively throughout the incubation period (Figure 7A,B). The inhibition rate increased from 3.67 ± 1.65% on day 1 to 24.95 ± 2.45%, 42.46 ± 1.23%, and 54.13 ± 1.23% on days 2, 3, and 4, respectively. After day 5, mycelial growth of treatments were not observed, and the inhibition rate reached a maximum of 60.52 ± 2.00% (Figure 7C). The inhibitory effect of SWFU41 increased significantly over time (*p* < 0.05), indicating its potential as a biological control agent against *P. microspora*.

## 4. Discussion

Leaf spot disease caused by *P. microspora* is the predominant foliar disease affecting *A. villosum* cultivation in Wenshan, Yunnan, China. Field surveys revealed an incidence rate of up to 56% in commercial plantations. Lesions initially appear as irregular, greyish-white spots with brown margins as infection progresses, black spots become partially embedded in the leaf surface. These symptoms damage leaf structure and impair photosynthesis, severely reducing yield and fruit set, compromising fruit quality and medicinal properties, and causing substantial economic losses to growers. *Pestalotiopsis* spp. are among the primary pathogens causing leaf spot diseases in plants, with a wide host range affecting numerous plants. Currently reported diseases caused by *Pestalotiopsis* sp. pathogens include cocoa leaf spot [41], guava scab disease [42], coconut leaf spot [43], bayberry twig blight [44], dracaena dead branch disease [45], mango fruit rot [46], grape leaf spot, and post-harvest fruit rot [47]. Specifically, *P. virgatula* causes fruit rot in rambutan in the Hawaii region [48]; Hawaiian nut leaf blight is caused by *P. colombiensis* in the Hawaiian nut-growing region of Pu’er, Yunnan, China [49]; leaf spot disease of evening primrose (*Oenothera laciniata* Hill.) is caused by *P. oenotherae* [50]; grey mold of *Camellia sinensis* (tea) is caused by *P. furcata* [15]; and *P. sydowiana* causes root, stem and leaf disease leading to death in potted rhododendron [51]. Certain fruit ulcers and stem blights are caused by *P. clavispora* and *P. neglecta* [19]. This study is the first to report the pathogenic characteristics of *P. microspora* associated with leaf spot disease of *A. villosum*. This finding expands the known host range of *Pestalotiopsis* spp. and contributes to a better understanding of pathogen–host associations within this genus. Furthermore, the antagonistic activities of *Trichoderma* spp. and *B. velezensis* SWFU41 against *P. microspora* were evaluated under in vitro conditions, providing preliminary evidence for their potential application in the management of *A. villosum* leaf spot disease.

As an important plant pathogenic fungus, *P. microspora* has been the subject of significant research. Complete genome sequencing of this pathogen revealed a genome size of approximately 50.31 Mb and an overall GC content of 50.25% [52]; *P. microspora* has a broad host range, including *Actinidia chinensis* [53], *Ampelopsis grossedentata* [54], *Eriobotrya japonica* [55], *Persea americana* [56], *Psidium guajava* [57] and *Vaccinium corymbosum* [58]. This study is the first to report the infection of *A. villosum* leaves by *P. microspora*, providing a foundation for further research on the host range and life history of this pathogen. Biological control of *P. microspora* is still limited to the laboratory. *Allium sativum*, *Syzygium aromaticum*, and *Zingiber officinale* have been shown to inhibit the in vitro growth and conidial germination of *P. microspora* [59,60]. This study confirmed that *Trichoderma* spp. (*T. harzianum* and *T. asperellum*) and *B. velezensis* SWFU41 exerted a significant inhibitory effect on *P. microspora*, providing a new strategy for the biological control of this disease.

In this study, the antagonistic activities of two *Trichoderma* spp. (*T. asperellum* and *T. harzianum*) and *B. velezensis* SWFU41 against *P. microspora* were systematically evaluated using a dual-plate confrontation assay. The results demonstrated that all three biocontrol agents exhibited antifungal activity against *P. microspora*, although their inhibitory efficiencies varied. Based on the inhibition rate, *B. velezensis* SWFU41 showed a higher inhibitory level than the tested *Trichoderma* strains under the experimental conditions, with complete inhibition of fungal growth observed at 5 days. Previous studies have shown that *B. velezensis* can suppress fungal pathogens through the production of antimicrobial metabolites, including lipopeptides such as surfactin, fengycin, and iturin, which may interfere with fungal cellular structures and physiological functions [61]. This finding may partly explain the rapid inhibitory effects observed for strain SWFU41 during the early stages (1–4 days) of the confrontation assay. Furthermore, the cessation of pathogen growth after 5 days suggests that SWFU41 may inhibit *P. microspora* through one or more antagonistic mechanisms, which require further investigation. Gao et al. reported that lipopeptides produced by *B. velezensis* G-1 showed strong antifungal activity against *Alternaria* spp., resulting in over 80% inhibition and inhibition zones exceeding 25 mm [62]. In the present study, the inhibition rate of *B. velezensis* SWFU41 against *P. microspora* was 60.52% under the evaluated conditions. Although both strains belong to the same species, the inhibitory activity of SWFU41 was lower than that reported for G-1 under their respective experimental conditions. This difference may reflect variation in strain-specific antimicrobial capacity and differences in pathogen susceptibility among fungal genera.

Although the initial inhibition rate of *T. asperellum* was lower than that of strain SWFU41, it fully colonized the culture plate by 7 d. Card et al. also demonstrated in their study on strawberry gray mold control that *T. asperellum* LU132 effectively inhibited mycelial growth and sporulation of the pathogen, attributing this efficacy to the combined effects of nutrient competition, the production of non-volatile metabolites, and mycoparasitism [63]. The difference in inhibitory efficacy between *T. asperellum* and *T. harzianum* observed in this study indicates that biocontrol potential varies among different species within the same genus. Regarding the combined application of *Bacillus* spp. and *Trichoderma* spp., previous studies have shown that the co-application of *B. velezensis* and *Trichoderma* spp. achieved better control of tomato wilt than either agent alone [64]. Therefore, future research should further explore combined application strategies for *B. velezensis* and *Trichoderma* spp. However, attention must be paid to the potential antagonism between them, and approaches such as strain screening or genetic modification should be employed to convert antagonism into synergy, thereby facilitating the development of a composite biocontrol agent that combines rapid and long-lasting effects. However, with rising consumer demands for food safety and quality, coupled with increasingly stringent quarantine standards for traditional Chinese medicinal products in China, the market demand for high-quality *A. villosum* continues to grow. Therefore, controlling leaf spot disease is crucial for ensuring plant health and enhancing fruit quality and yield. Conducting research on biological control of *P. microspora* has become a key direction for promoting the green and sustainable development of the *A. villosum* industry. Our research team will continue to study prevention and control technologies for leaf spot disease caused by *P. microspora*, further refine the theoretical framework of plant pathology, and provide reference for other fungal diseases.

## 5. Conclusions

Leaf spot disease of *A. villosum* was investigated in Wenshan, Yunnan Province, China. Based on symptom observation, morphological characterization, multilocus phylogenetic analyses (*ITS*, *LSU*, and *TUB*), and pathogenicity tests, the causal agent was identified as *P. microspora*. To our knowledge, this is the first report of *P. microspora* causing leaf spot disease on *A. villosum*. Koch’s postulates were fulfilled by re-isolation of the pathogen from inoculated leaves. Among the tested biocontrol microorganisms, *T. asperellum* and *T. harzianum* exhibited antagonistic activity against *P. microspora*, with maximum inhibition rates of 50.70% and 44.77%, respectively, under in vitro conditions. The endophytic bacterial strain SWFU41, isolated from healthy *A. villosum* leaves and identified as *B. velezensis* based on morphological and molecular evidence, showed the highest inhibition rate among the tested strains, reaching 60.52% under in vitro conditions. These findings provide a scientific basis for disease diagnosis and epidemiological investigations of *A. villosum* leaf spot disease. In addition, the tested biocontrol agents showed inhibitory activity against *P. microspora* under in vitro conditions; however, their efficacy under greenhouse and field conditions remains to be further evaluated.

## Figures and Tables

**Table 1:** Primer Sequences of this study.

Gene	Primer	Sequence (5′–3′)	Annealing Temperature (°C)	Reference
*LSU*	LR5	ATCCTGAGGGAAACTTC	51	[31]
	LROR	GTACCCGCTGAACTTAAGC		[32]
*ITS*	ITS1	TCCGTAGGTGAACCTGCGG	60	[33]
	ITS4	TCCTCCGCTTATTGATATGC		
*TUB*	BT2a	GGTAACCAAATCGGTGCTGCTTTC	62	[34]
	BT2b	ACCCTCAGTGTAGTGACCCTTGGC		
16S rRNA	B27F	AGAGTTTGATCCTGGCTCAG	49	[35]
	1492R	TACCTTGTTACGACTT		
*gyrB*	UP1	GAAGTCATCATGACCGTTCTGCAYGCNGGNGGNAARTTYGA	73	[36]
	UP2r	AGCAGGGTACGGATGTGCGAGCCRTCNACRTCNGCRTCNGTCAT		

**Table 2:** Fungal GenBank accession numbers used in the phylogenetic analyses.

Species and Strain	Locality, Host/Substrate	Gen Bank Accession Numbers		
		*ITS*	*LSU*	*TUB*
*Pestalotiopsis microspora*				
**SWFU30**	**China; *Amomum villosum***	**PX806344**	**PX806352**	**PZ224677**
**SWFU31**	**China; *Amomum villosum***	**PX810979**	**PX810981**	**PZ224678**
CWK1114, YBLJ-1	China; *Homo* sapiens, *Cathaya argyrophylla*	KY366172	KY366173	OQ693711
AV-157	India; Avocado	PQ309015	PQ299658	PQ801745
*P. telopeae*				
CBS 113606	Australia; *Telopea* sp.	KM199295	KM116202	KM199402
CBS 114137	Australia; *Protea susannae* cv. Pink Ice	KM199301	KM116219	KM199469
*P. australasiae*				
CBS 114126	New Zealand; *Knightia* sp.	KM199297	KM116218	KM199409
CBS 114141	Australia; *Protea* sp.	KM199298	KM116203	KM199410
*P. camelliae*				
CBS 443.62	Turkey; *Camellia sinensis*	KM199336	KM116225	KM199424
*P. oryzae*				
CBS 353.69	Denmark; *Oryza sativa*	KM199299	KM116221	KM199398
CBS 171.26	Italy	KM199304	KM116206	KM199397
*P. papuana*				
CBS 331.96	Papua New Guinea; *Coastal soil*	KM199321	KM116240	KM199413
CBS 887.96	Papua New Guinea; *Cocos nucifera*	KM199318	KM116231	KM199415
*P. australis*				
CBS 111503	South Africa; *Protea susannae*	KM199331	KM116200	KM199382
CBS 114474	South Africa; *P. susannae*	KM199334	KM116220	KM199385
*P. chamaeropis*				
CBS 186.71	Italy; *Chamaerops humilis*	KM199326	KM116210	KM199391
CBS 237.38	Italy	KM199324	KM116217	KM199392
*P. diploclisiae*				
CBS 115449	China; *Psychotria tutcheri*	KM199314	KM116215	KM199416
CBS 115587	China; *Diploclisia glaucescens*	KM199320	KM116242	KM199419
*P. humus*				
CBS 115450	Hong Kong; *Ilex cinerea*	KM199319	KM116208	KM199418
CBS 336.97	Papua New Guinea; Soil	KM199317	KM116230	KM199420
*P. kenyana*				
CBS 442.67	Kenya; *Coffea* sp.	KM199302	KM116234	KM199395
CBS 911.96	Raw material from agar-agar	KM199303	KM116204	KM199396
*P. knightiae*				
CBS 111963	New Zealand; *Knightia* sp.	KM199311	KM116241	KM199406
CBS 114138	New Zealand; *Knightia* sp.	KM199310	KM116227	KM199408
*P. monochaeta*				
CBS 144.97	Netherlands; *Quercus robur*	KM199327	KM116229	KM199386
CBS 440.83	Netherlands; *Taxus baccata*	KM199329	KM116196	KM199387
*P. parva*				
CBS 265.37	*Delonix regia*	KM199312	KM116226	KM199404
CBS 278.35	*Leucothoe fontanesiana*	KM199313	KM116205	KM199405
*P. adusta*				
MFLUCC10-0146; 3.9098	Thailand, China; *Syzygium* sp.	JX399007	JN940825	JX399038
*P. biciliata*				
CBS 790.68	Netherlands; *Taxus baccata*	KM199305	KM116235	KM199400
*Boeremia exigua*				
BZ2	China; tobacco	PP917553	PP922345	PP932497

**Note:** The boldface entry denotes the strain isolated in this study.

**Table 3:** Inhibition of *P. microspora* by *T. asperellum* and *T. harzianum*.

Strain	3 d	4 d	5 d	6 d	7 d
*T. asperellum*	7.76% ± 2.10 c	27.61% ± 6.58 bc	36.85% ± 2.88 ab	43.71% ± 2.10 a	50.70% ± 2.41 a
*T. harzianum*	7.02% ± 3.04 c	21.54% ± 7.56 b	32.57% ± 2.83 b	38.30% ± 0.72 a	44.77% ± 3.58 a

Different letters within the row represent significant differences (*p* < 0.05).

**Table 4:** Bacterial GenBank accession numbers used in the phylogenetic analyses.

Species and Strain	Gen Bank Accession Numbers	
	16sRNA	*gyrB*
*Bacillus velezensis*		
**SWFU41**	**PZ498242**	**PZ498543**
DH82	MK203035	MK203036
JM-3	MG841149	MT634153
hjt6	OR264129	OR327565
*B. amyloliquefaciens*		
BCRC 14193	EF433408	DQ309309
BCRC 17038	DQ993675	DQ309305
*B. atrophaeus*		
BCRC 17123	EF433409	DQ309296
BCRC 17530	DQ993677	DQ309302
*B. licheniformis*		
BCRC 11702	EF433410	DQ309295
BCRC 12826	EF423608	DQ309323
BCRC 14353	EF423609	DQ309324
BCRC 15413	DQ993676	DQ309325
*B. mojavensis*		
BCRC 17124	EF433405	DQ309297
BCRC 17531	DQ993678	DQ309303
*B. sonorensis*		
BCRC 17416	EF433411	DQ309300
BCRC 17532	DQ993679	DQ309304
*B. subtilis*		
BCRC 14716	EF423595	DQ309314
BCRC 17435	EF423598	DQ309317
H158	MT348555	MT359893
*B. cereus*		
XWH120710-1	KF022230	KF022229
XWH120710-2	KF022231	KF022228
*Paenibacillus polymyxa*		
IAM 13419	NR_037006	AB464839

**Note:** The boldface entry denotes the strain isolated in this study.

## Data Availability

The original contributions presented in this study are included in the article. Further inquiries can be directed to the corresponding authors.
